# Subjective age of acquisition norms for 1604 English words by Spanish L2 speakers of English and their relationship with lexico-semantic, affective, sociolinguistic and proficiency variables

**DOI:** 10.3758/s13428-022-02026-9

**Published:** 2022-12-07

**Authors:** Sara Rodriguez-Cuadrado, José Antonio Hinojosa, Marc Guasch, Carlos Romero-Rivas, Lucía Sabater, Paz Suárez-Coalla, Pilar Ferré

**Affiliations:** 1 Departamento Interfacultativo de Psicología Evolutiva y de la Educación, Facultad de Formación del Profesorado y Educación, Universidad Autónoma de Madrid, C/ Francisco Tomás y Valiente, n° 3, 28049 Madrid, Spain; 2 Instituto Pluridisciplinar, Universidad Complutense de Madrid, Madrid, Spain; 3 Departamento Psicología Experimental, Procesos Cognitivos y Logopedia, Universidad Complutense de Madrid, Madrid, Spain; 4 Centro de Investigación Nebrija en Cognición (CINC), Universidad Nebrija, Madrid, Spain; 5 Departamento de Psicología y CRAMC, Universitat Rovira i Virgili, Tarragona, Spain; 6 Departamento de Psicología y Grupo de Investigación INCO, Universidad de Oviedo, Oviedo, Spain

**Keywords:** Age of acquisition, Second language speakers, Boosted regression trees

## Abstract

Psycholinguistic studies have shown that there are many variables implicated in language comprehension and production. At the lexical level, subjective age of acquisition (AoA), the estimate of the age at which a word is acquired, is key for stimuli selection in psycholinguistic studies. AoA databases in English are often used when testing a variety of phenomena in second language (L2) speakers of English. However, these have limitations, as the norms are not provided by the target population (L2 speakers of English) but by native English speakers. In this study, we asked native Spanish L2 speakers of English to provide subjective AoA ratings for 1604 English words, and investigated whether factors related to 14 lexico-semantic and affective variables, both in Spanish and English, and to the speakers’ profile (i.e., sociolinguistic variables and L2 proficiency), were related to the L2 AoA ratings. We used boosted regression trees, an advanced form of regression analysis based on machine learning and boosting algorithms, to analyse the data. Our results showed that the model accounted for a relevant proportion of deviance (58.56%), with the English AoA provided by native English speakers being the strongest predictor for L2 AoA. Additionally, L2 AoA correlated with L2 reaction times. Our database is a useful tool for the research community running psycholinguistic studies in L2 speakers of English. It adds knowledge about which factors—linked to the characteristics of both the linguistic stimuli and the speakers—affect L2 subjective AoA. The database and the data can be downloaded from: https://osf.io/gr8xd/?view_only=73b01dccbedb4d7897c8d104d3d68c46.

## Introduction

Research on bilingualism has acquired a central role in psycholinguistics in recent decades. One of the main research objectives has been to establish the distinctive characteristics of language processing in bilinguals. To that end, many studies have examined bilinguals’ performance in tasks including second language (L2) words, sentences or longer utterances, and compared them, in some cases, to first language (L1) units (e.g., Ferré et al., 2017; Whitford & Titone, 2017). To select the experimental materials for psycholinguistic studies, it is necessary to consider several lexico-semantic and affective variables which are known to influence word processing, such as word frequency, familiarity, concreteness or valence, among others. A common practice in bilingualism research is to obtain the values for these variables from word ratings provided by native speakers. That is, L2 speakers are presented in most studies with L2 materials rated by L1 speakers, which limits our understanding of the influence of those lexico-semantic and affective variables and potentially jeopardises the validity of the research findings. Using data provided by L2 speakers could make L2 research more ecologically valid and provide further insight into L2 acquisition and processing. However, only a few studies so far have gathered ratings from L2 speakers for distinct lexico-semantic and affective variables. For instance, Chen and Dong (2019) collected subjective frequency ratings for English words in a sample of Chinese L2 English learners and compared them to objective frequency ratings obtained from six English corpora. Interestingly, a superiority for subjective L2 frequency ratings over objective ratings in predicting L2 lexical processing was observed. Considering these findings, the authors recommended that researchers collect subjective frequency estimates from L2 learners in studies about L2 processing. A similar conclusion was reached by Wang and Chen (2020), who collected familiarity ratings for English words from Chinese-English bilinguals and found only a moderate correlation between these ratings and objective frequency measures obtained from English film subtitles (SUBTLEX-UK, van Heuven et al., 2014). Similarly, in the study of Hubers et al. (2020), German L2 learners of Dutch rated Dutch idioms on frequency of exposure and frequency of use, meaning familiarity, imageability and transparency, finding that non-native speakers’ intuition regarding L2 idioms was a reliable source of information (see also Hasegawa, 2010, for L2 imageability).

Another set of studies, focused on affective features, collected valence ratings in L2 speakers of English who had a variety of L1s (Ferré et al., 2022a, Garrido & Prada, 2021, Imbault et al., 2021, Vélez-Uribe & Rosselli, 2019). All of them found more attenuated affective ratings in the L2 than in the L1. In addition, Imbault et al. (2021) showed that ratings were modulated by the characteristics of the words and the speakers. In particular, they found more native-like ratings for high-frequency words and by more proficient L2 speakers who had lived longer in the L2 country. Similarly, the L2 experience of the speakers (proficiency and age and context of L2 acquisition) was reported to modulate familiarity ratings of L2 words in the study by Garrido and Prada (2021).

Another variable that has attracted the attention of researchers on bilingualism is the age of acquisition (AoA) of words. AoA is the age at which a word was acquired, and there are two basic methods used to estimate it: an objective method, based on studies on children (e.g., asking children of different ages to name pictures; see Morrison et al., 1997), and a subjective method, based on adults’ estimations of the age at which they acquired the words. Several studies have found a high correlation between AoA values obtained from both methods (e.g., Chalard et al., 2003; Liu et al., 2011; Morrison et al., 1997; Walley & Metsala, 1992). AoA is a variable that can help researchers understand the links between orthography, phonology and semantics in the lexicon (Juhasz, 2005). In fact, the use of AoA (either manipulating it or controlling it across experimental conditions) is quite common in psycholinguistic research. The “age of acquisition effect” reflects how words with an early AoA are recognised faster and/or more accurately than those with a late AoA (e.g., Cortese & Khanna, 2007; Juhasz & Rayner, 2006; Kuperman et al., 2012, 2014; Sereno & O’Donnell, 2009; for a review see Johnston & Barry, 2006). Most research investigating AoA has relied on data from normative studies to obtain the experimental materials. A few databases are available on both objective and subjective AoA in a variety of languages (e.g., Barca et al., 2002, in Italian; Cameirao & Vicente, 2010, in Portuguese; Ferrand et al., 2008, in French; Kuperman et al., 2012, in English; Liu et al., 2007, in Chinese; Moors et al., 2013, in Dutch; Schröder et al., 2012, in German; Alonso et al., 2015; Cuetos et al., 1999; Hinojosa et al., 2016b, and Piñeiro & Manzano, 2000, in Spanish; see also Łuniewska et al., 2016, 2019, for studies that have compared AoA on a total of 32 languages). All these normative studies provide AoA ratings collected from native speakers. However, the AoA of words in an L2 may also be a relevant variable in explaining bilingual language processing.

Thus, to explore L2 vocabulary acquisition, an essential stepping stone would be to elucidate the age at which L2 speakers acquire certain words, and the variables that influence this acquisition. To enable such research, normative AoA ratings for L2 words would be highly valuable. Izura and Ellis (2002 obtained L2 AoA ratings, but for a small set of items. To our knowledge, only two studies have collected this kind of rating for a large set of words, gathering subjective AoA ratings for a set of English words from L2 speakers (unbalanced late bilinguals): Dutch-English bilinguals in the study of Dirix and Duyck (2017), and Chinese-English bilinguals in the study of Wang and Chen (2020). These studies reported a relevant effect of the AoA of L2 words in language processing. Concretely, Dirix and Duyck (2017) found that L2 AoA ratings modulated eye movements during reading. Wang and Chen (2020), in turn, demonstrated that L2 AoA could account for an additional part of the variance on lexical decision times of L2 speakers of English (Berger et al., 2019) once other relevant variables had been controlled for. Finally, Izura and Ellis (2002) found that response times (RTs) in a lexical decision task performed in the participants’ L2 were predicted by L2 AoA, but not by L1 AoA. These authors concluded that L2 AoA effects reflect the order in which the words were acquired in L2, rather than the order in which their translation equivalents were acquired in the native language. In turn, this would suggest that the AoA effect is not related to the acquisition of words’ meanings, but rather to the acquisition of word forms or the mappings between lexical and semantic representations (that is, in line with the *mapping hypothesis*, see also Cortese & Schock, 2013).

However, the aforementioned databases have not explored in depth the role of (and the relationship amongst) lexico-semantic and affective variables in subjective L2 AoA, nor have they carefully examined speaker-related variables such as the role of proficiency and linguistic history relative to these ratings. In addition, to our knowledge, no database has gathered English AoA ratings provided by late unbalanced Spanish L2 speakers of English, so the present database could be widely used by those researchers working with this population.

In the current study we examined the relationship between subjective AoA in an L2 (in this case, English) with different lexico-semantic and affective variables that impact word processing and are related to AoA. We also considered the participants’ sociolinguistic background and measured their L2 proficiency. Regarding lexico-semantic and affective variables, we included concreteness, frequency, familiarity, prevalence, word length, number of orthographic neighbours, iconicity, imageability, sensory experience ratings, semantic size, cognate status, valence and arousal, along with AoA ratings for English and Spanish.

A number of these variables correlate with AoA in L1. Regarding *concreteness* (that is, the extent to which something can be experienced through our senses), concrete words are acquired earlier (e.g., Morris, 1981). As for *frequency* (meaning how often a word is found, mostly in print), high *frequency* words have an earlier acquisition (e.g., Citron et al., 2014), and words acquired earlier are also more *familiar* (familiarity being a measure of a person’s experience with a word; see Hinojosa et al., 2016b).^1^ Also, words with a low AoA are more *prevalent* (“prevalence” being a measure of a population’s word knowledge; Brysbaert & New, 2009, Brysbaert et al., 2016). *Iconicity* (the resemblance between the form and the meaning of a word) helps language acquisition (Imai & Kita, 2014; Massaro & Perlman, 2017; Perniss & Vigliocco, 2014) and is related to AoA (Perry et al., 2015), so words with high iconicity scores are acquired earlier and are more frequent in infancy than words with low iconicity scores in both oral and sign languages (Caselli & Pyers, 2017; Hinojosa et al., 2021; Sidhu et al., 2021; Thompson et al., 2012; Vinson et al., 2008). In addition, *imageability* (a measure of how easy it is for a person to create a mental image of something) scores are higher for words with early AoA (Citron et al., 2014). Words with high *sensory experience ratings* (SERs, a measure of how much a word generates a sensory experience in the mind of a person) are acquired earlier (see Hinojosa et al., 2016b^2^). *Semantic size* (a measure of magnitude; e.g., big, small) also correlates positively with AoA, as words with high semantic size are learned earlier (Scott et al., 2019). In addition, *cognate status* (i.e., the degree of orthographic overlap between an L2 word and its L1 translation equivalent) influences L2 word acquisition, where cognates are learned sooner than non-cognates (see Tonzar et al., 2009, and Comesaña et al., 2012 for data on children). Regarding *valence* (a measure of a word’s hedonic positive or negative value) and *arousal* (a measure of the internal activation elicited by a word), words with an early AoA tend to be mostly pleasant and calm (Citron et al., 2014; Hinojosa et al., 2016b; Warriner et al., 2013). In addition, a variable that correlates with L2 AoA is L2 familiarity. The study of Wang and Chen (2020) shows an interaction between L2 familiarity and L2 AoA, where those words which were less familiar in the L2 were also acquired later in the L2. A positive correlation between L1 and L2 AoA was also found.

Many of the aforementioned lexico-semantic variables modulate word processing, as AoA does (e.g., Brysbaert et al., 2016; Dijksterhuis & Aarts, 2003; Ferré et al., 2018; Hinojosa et al., 2010, 2020; Imai et al., 2008; Juhasz & Rayner, 2003; Kantartzis et al., 2011; Kousta et al., 2009; Kuperman et al., 2012; Peeters, 2016; Scott et al., 2009, 2019; Sereno et al., 2009; Sidhu et al., 2020; Winter et al., 2017; Yao et al., 2013). In addition, it is important to consider other relevant variables in word processing such as *word length* (which positively influences processing time; see Davies et al., 2013 and Kuperman et al., 2012), or *orthographic neighbourhood size* (the number of similarly spelled words), which, in turn, is related to psycholinguistic variables such as word frequency (e.g., Grainger, 1990; Yarkoni et al., 2008). Words from sparse neighbourhoods are benefitted in recognition, naming and lexical decision tasks (for developmental differences, see Garlock et al., 2001). Finally, the studies that have examined the effect of *semantic size* show that words referring to bigger things benefit from faster recognition (Scott et al., 2019; Sereno et al., 2009; Yao et al., 2013).

The influence of all these variables on both L1 and L2 processing has been notorious (e.g., van Heuven et al., 1998), where cognate status greatly modulates these effects, facilitating L2 processing (e.g., Costa et al., 2000). Given the relevance of these variables on the study of L1 and L2 processing, and their relationship with AoA in the L1, we include them in our study to better describe and understand subjective AoA ratings in L2, specifically those of late unbalanced L2 Spanish- English bilinguals. In addition, we examine the relationship between the above-mentioned variables and ratings of AoA in the L2 (English) considering, when available, the ratings of these variables both in L1 (Spanish) and in L2 (English).

It should be noted that, although there are two previous studies gathering L2 AoA ratings (Dirix & Duyck, 2017; Wang & Chen, 2020), they did not take into consideration the sociolinguistic characteristics of their participants. In this study, however, we examine several subjective and objective measures of the participants’ linguistic history and proficiency. Specifically, we have included a sociolinguistic survey (see Materials and Procedure) and have objectively measured the participants’ English proficiency with the LexTALE English test (Lexical Test for Advanced Learners of English; Lemhöfer & Broersma, 2012), given that variables related with the speakers such as proficiency and language use, among others, can influence how lexico-semantic (Guasch et al., 2008; Perani & Abutalebi, 2005) and affective (Degner et al., 2011) variables impact L1/L2 language processing.

In sum, knowing the L2 AoA of words would allow researchers to select those stimuli that participants are likely to know (or not know, depending on the research purposes). Having an appropriately normed dataset on AoA *by* and *for* L2 speakers fills a gap in the field. Also, exploring how L2 AoA is influenced by lexico-semantic and affective variables, and sociolinguistic and proficiency variables, is not only informative by itself, but can have relevant educational implications in the context of English as a foreign language (EFL) and bilingual education programmes (BEP). For instance, in the 2019/2020 academic year, the Spanish-English BEP was present in 50% of public schools, 59.2% of high schools and 54.7% of charter schools (Mañas Antón, 2019).

In addition, Izura and Ellis (2002) investigated (with a limited set of words) the locus of the AoA effect, by looking at whether L2 reaction times (RTs) could be predicted by AoA values in the first or second language of bilingual speakers. We aim at expanding this investigation with our L2 AoA data and L2 RTs from a previously published study (Berger et al., 2019).

### Objectives and hypotheses

Our first objective is to gather subjective AoA data for 1604 English words provided by Spanish L2 speakers of English. Our second objective is to examine if, and how, several variables related to the words, both lexico-semantic and affective, influence the ratings provided by Spanish L2 speakers of English. Thirdly, we will explore if, and how, sociolinguistic and proficiency variables related to the speakers influence these AoA L2 ratings. Finally, we will look at the relationship between AoA and L2 processing with L2 RTs. Accordingly, and based on previous literature, we derive the following hypotheses linked to our second and third objectives: Regarding the second objective, we expect a positive relationship between L1 (Spanish) AoA and L2 AoA scores (Dirix & Duyck, 2017; Wang & Chen, 2020). In addition, and in line with Wang and Chen (2020), we expect to find a negative relationship between L2 familiarity (that is, familiarity scores in English) and L2 AoA, so more familiar words would be acquired earlier. With respect to the third objective, we are not knowledgeable of any study that has looked at sociolinguistic variables in detail when examining AoA. However, previous studies have shown a relationship between several sociolinguistic factors and lexico-semantic and/or affective variables in L2. For instance, Imbault et al. (2021) found that L2 speakers had greater word knowledge and provided more native-like ratings for valence and arousal when they learned English at a younger age, for more years, in an immersive context, had a high self-reported proficiency, and used English frequently in their day-to-day lives. Therefore, we expect an effect of those variables on L2 AoA ratings. Finally, following Izura and Ellis (2002), we hypothesise that what matters for RTs in an L2 lexical decision is the L2 AoA of the L2 words, but not the AoA in Spanish (the participants’ L1) of the translation equivalents, which will speak in favour of the *mapping hypothesis* and against a semantic locus in AoA effects.

## Methods

### Participants

A total of 309 participants took part in the study. After data trimming (see “Results”), the final group consisted of 292 participants (242 women, 50 men) with a mean age of 20.91 years (*SD* = 3.18; range = 18–39), and who were recruited from several Spanish universities, including Universidad de Oviedo (41%), Universitat Rovira i Virgili (26%), Universidad Complutense de Madrid (23%) and other universities (10%).

After completing the AoA task (see below), participants took the LexTALE English test (Lemhöfer & Broersma, 2012) and filled out a sociolinguistic survey (see “Materials and procedure”). Their mean score in the LexTALE test was 67.02 (*SD* = 9.22; range = 50–90). Following the criteria established by Lemhöfer and Broersma with a Dutch sample (2012), advanced users (C1–C2) would score between 80 and 100, upper intermediate users (B2) would score between 60 and 79, and lower intermediate (B1) and basic (A2) users would score 59 or less (Lemhöfer & Broersma, 2012, p. 341). Therefore, based on Lemhöfer and Broersma (2012), the average English level of our participants could be classified as upper intermediate.

Responses to the sociolinguistic questionnaire revealed that all participants were native Spanish speakers or bilingual in Spanish and one of the other official languages in Spain (i.e., Basque, Catalan or Galician); 99.31% of the participants had completed at least a baccalaureate level of studies, professional training or higher, and 38.01% had completed a university degree or higher (e.g., masters, PhD). The contexts in which participants reported having learned English were mainly at school (94.86%) and informal settings (41.10%; i.e., with friends, films or music). Only 5.82% of the participants reported having learned English in an English-speaking country, where they had lived for less than 1 year on average. Participants reported spending an average of 13.92 years (*SD* = 3.66; range = 3–25) learning English. They reported using English 23.95% (*SD* = 21.23; range = 0–90) of the time in a week. Table 1 shows their mean estimated AoA of English and their self-rated proficiency in English in the four basic linguistic skills.

**Table 1:** Mean and standard deviation (in parentheses) of age of acquisition and self-rated proficiency in English of the participants

Age of acquisition of English (in years)				
Speaking	Reading	Writing	Listening	Mean
8.00 (4.11)	7.72 (3.05)	8.13 (3.25)	7.30 (3.88)	**7.79 (3.61)**
Self-rated English proficiency (1–7 scale; 1 = very bad, 7 = very good)				
Speaking	Reading	Writing	Listening	Mean
4.51 (1.46)	5.36 (1.27)	4.62 (1.32)	4.98 (1.50)	**4.87 (1.43)**

Bold indicates mean of four linguistic skills

All participants accepted an informed consent form in Spanish before starting the study, which was conducted with the approval of the Ethics Committee of the Principality of Asturias (reference 153/19). The study was performed in accordance with the ethical standards as laid down in the 1964 Declaration of Helsinki.

## Materials and procedure

Words were selected considering the AoA values of normative studies developed in English (Scott et al., 2019) and Spanish (Sabater et al., 2020). We firstly selected a large set of words (*n* = 1258) with an AoA equal to or below 7 years of age, to be sure that the participants were likely to know them. The selection was completed with 346 additional words with an AoA above 7, resulting in a total of 1604 words.

The words were randomly divided into 11 lists, nine containing 146 words and two containing 145 words. Each list was rated by a minimum of 25 participants (*M* = 26.55, *SD* = 1.21, range = 25–28).

**Fig. 1:**
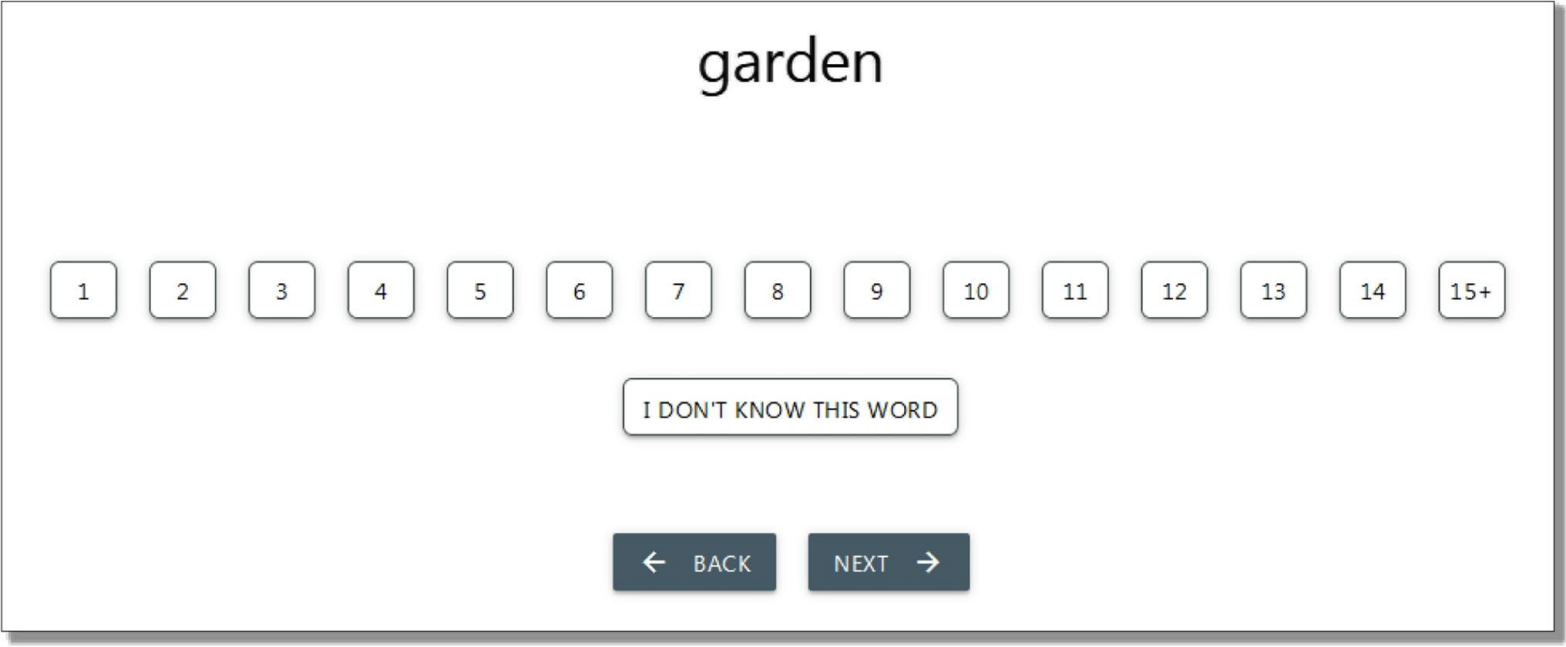
Layout of a word and the rating scale in the age of acquisition rating task

A single questionnaire was designed in web format and was administered online with a unique URL that randomly fed from one of the 11 lists at each access. The questionnaire started with an information screen in which participants had to provide their explicit consent to participate by ticking a box. Next, participants were asked to provide their home university, age and gender. These first two screens were presented in Spanish to ensure understandability. Then, the task instructions were displayed (in English) as follows: “*A word’s age of acquisition is the age at which that word was initially learned. Please estimate when in your life you think you first acquired or learned each of the presented words. That is, try to remember how old you were when you learned each word either in its spoken or written form (whichever came first). We mean the age at which you would have understood that word if somebody had used it in front of you, EVEN IF YOU DID NOT use, read, or write it at the time. In order to indicate the estimated age of acquisition, please click in one of the following boxes:*”. Next, an image of the response scale was presented (see Fig. 1), followed by the reminder: “*Please, remember that you are not being asked about the age at which you acquired the word in Spanish, but the age at which you think you acquired the word in English.*”

After displaying the instructions, the words from the selected list were presented randomly, one at a time, following the layout of Fig. 1 with a progress bar at the bottom. A response to each word was required to advance throughout the task, but participants had the option to mark a word as unknown.

After the ratings, the English version of the LexTALE vocabulary test (Lemhöfer & Broersma, 2012) was administered, closely following the implementation and scoring system proposed on the LexTALE website (www.lextale.com). It is composed of 60 trials and takes less than 5 minutes to complete. Participants received feedback on their scores immediately after completing the test.

In the final part of the questionnaire, participants were asked in Spanish to answer some questions about their sociolinguistic background. This section was divided into three screens. In the first one, they were asked about their level of education, their mother tongue (where they could report a maximum of two languages), and if they have or had any type of difficulty affecting reading and/or writing (and which one). On the next screen they were asked about the age at which they had acquired four basic skills in English, namely speaking, reading, writing and listening, and the number of years they had been studying English and the context(s) in which they had acquired English (i.e., at home, at school, informally [i.e., with friends, through films or music], living in an English-speaking country, or other). If they ticked the option of having lived in an English-speaking country, they were asked about which country and the time they had spent living there. They were also asked to rate their proficiency level on a 7-point scale (1 = very bad, 7 = very good) for each of the four basic skills—speaking, reading, writing and listening—in English. Finally, on the last screen before submitting the information, they were asked to estimate the percentage of time they used English over the course of a week and could add clarifying comments if they so wished.

The average time to complete the whole task was around 20 minutes. Each participant rated only one list of words and did not complete the questionnaire more than once.

Finally, to explore the relationship between L2 AoA values and L2 RTs in native Spanish L2 speakers of English, we extracted RT data from Berger et al. (2019). Out of their 1315 non-native speakers, 532 (40%) were dominant in Spanish, where we shared 1089 words between databases.

## Results

### Availability of the norms

The ratings are available at https://osf.io/gr8xd/?view_only=73b01dccbedb4d7897c8d104d3d68c46 under the name “Subjective age of acquisition norms for 1604 English words by Spanish L2 speakers of English”. The datasheet contains the list of 1604 words rated in English together with their translation to Spanish, and the mean AoA (*Mean_AoA_Eng*) and their standard deviation (*SD_AoA_Eng*). For the calculation of other indicators (e.g., standard error), the number of people who rated each word is also given (*N*). However, not all words were known by all participants, so a column with the percentage of participants who knew each word is also included (*Perc_knowledge*).

A second file entitled “Lexico-semantic and affective values of the items” is also available at https://osf.io/gr8xd/?view_only=73b01dccbedb4d7897c8d104d3d68c46. This file has two sheets. The first sheet (entitled “Summary”) lists all the variables with their abbreviation (*Variable*), the *predictor* (e.g., “concreteness in English”), the number of words out of the 1,604 where the values associated to the variables were available (*Availability*) and the *source* (i.e., which database) from which we extracted the values of the variables. There is also a legend of sources with the abbreviated and full reference of the source (this is also available in Table 2). On the second sheet (entitled “Data”) the values for each variable associated to the word (when available) are listed. Please note that headings correspond to the variable name used on the first sheet.

**Table 2:** Predictors included in the study, classified by type of variable (lexico-semantic, affective, sociolinguistic or proficiency). Descriptions of the predictors and the abbreviations used in the analysis and graphs are provided, as well as the sources of the values and their relative contribution (RC) to the model (please note that if the predictor did not enter the simplified model, this is marked as n/a). Highest RCs are highlighted in bold.

Abbreviation	Predictor	Description	Source(s)	RC
Lexico-semantic variables				
eng_con	Concreteness in English	The extent to which an English word can be experienced by our senses	Scott et al., 2019	n/a
spa_con	Concreteness in Spanish	The extent to which the Spanish translation of the English word can be experienced by our senses	Duchon et al., 2013; Ferré et al., 2012; Guasch et al., 2016; Hinojosa et al., 2016b	1.4%
eng_zipf	Frequency in English	Word frequency in Zipf scale for the English word	Brysbaert & New, 2009	2.9%
spa_zipf	Frequency in Spanish	Word frequency in Zipf scale for the Spanish translation of the English word	Duchon et al., 2013	1.8%
eng_fam	Familiarity in English	Familiarity of the English word	Scott et al., 2019	**7.7%**
spa_fam	Familiarity in Spanish	Familiarity of the Spanish translation of the English word	Duchon et al., 2013; Ferré et al., 2012; Guasch et al., 2016; Hinojosa et al., 2016b	1.8%
eng_prevalence	Prevalence in English	A population’s knowledge of a word in English	Brysbaert et al., 2019	1.3%
spa_prevalence	Prevalence in Spanish	A population’s knowledge of the Spanish translation of the English word	Aguasvivas et al., 2018	1.6%
eng_length	Word length in English	Length in number of letters of the English word	n/a	n/a
spa_length	Word length in Spanish	Length in number of letters of the Spanish translation of the English word	n/a	n/a
eng_old20	Orthographic neighbours in English	Average Levenshtein distance of the 20 nearest neighbours in English	Balota et al., 2007	1.1%
spa_old20	Orthographic neighbours in Spanish	Average Levenshtein distance of the 20 nearest neighbours for the Spanish translation of the English word	Duchon et al., 2013	n/a
eng_ico	Iconicity in English	The resemblance between an English word and its meaning	Winter et al., 2022	1.1%
spa_ico	Iconicity in Spanish	The resemblance between the Spanish translation of the English word and its meaning	Hinojosa et al., 2021	n/a
eng_ima	Imageability in English	How easy it is to create a mental image of an English word	Scott et al., 2019	1.6%
spa_ima	Imageability in Spanish	How easy it is to create a mental image of the Spanish translation of the English word	Duchon et al., 2013; Guasch et al., 2016	1.6%
eng_ser	Sensory experience ratings in English	The extent to which an English word generates a sensory experience in the mind of a person	Juhasz & Yap, 2013	1.5%
spa_ser	Sensory experience ratings in Spanish	The extent to which the Spanish translation of the English word generates a sensory experience in the mind of a person	Díez-Álamo et al., 2019; Ferré et al., 2022b; Hinojosa et al., 2016b	1.4%
eng_size	Semantic size in English	Magnitude (big, small) of an English word	Scott et al., 2019	1.1%
Nld	Cognate status	Normalised Levenshtein distance between the English word and its translation in Spanish	Guasch et al., 2013	**5.1%**
eng_aoa	Age of acquisition in English	The age at which an English word is acquired	Scott et al., 2019	**14.3%**
spa_aoa	Age of acquisition in Spanish	The age at which the Spanish translation of the English word is acquired	Alonso et al., 2015	**4.7%**
Affective variables				
eng_val	Valence in English	An English word’s positive/negative value	Scott et al., 2019	1.1%
spa_val	Valence in Spanish	The Spanish translation of the English word’s positive/negative value	Ferré et al., 2012; Guasch et al., 2016; Hinojosa et al., 2016a; Stadthagen-Gonzalez et al., 2017	2%
eng_aro	Arousal in English	The internal activation provoked by an English word	Scott et al., 2019	1.1%
spa_aro	Arousal in Spanish	The internal activation provoked by the Spanish translation of the English word	Ferré et al., 2012; Guasch et al., 2016; Hinojosa et al., 2016a; Stadthagen-Gonzalez et al., 2017	n/a
Sociolinguistic variables				
age	Age	Age of the participant	n/a	2.3%
sex	Sex	Sex of the participant	n/a	n/a
education	Level of education	Level of education of the participant	n/a	n/a
difficulty	Reading or writing difficulty	If the participant had or has any reading or writing difficulty	n/a	n/a
age_s	Age of speaking in English	Age at which speaking in English was acquired	n/a	**4.2%**
age_r	Age of reading in English	Age at which reading in English was acquired	n/a	**4.2%**
age_w	Age of writing in English	Age at which writing in English was acquired	n/a	**7.2%**
age_l	Age of listening in English	Age at which listening in English was acquired	n/a	2.2%
years_e	Years studying English	Number of years that the participant has been studying English	n/a	**6.2%**
home	Context: home	If the participant acquired English at home	n/a	n/a
school	Context: school	If the participant acquired English at school	n/a	n/a
english_country	Context: English-speaking country	If the participant acquired English in an English-speaking country	n/a	n/a
informal	Context: Informal setting	If the participant acquired English informally (i.e., with friends, through films or music)	n/a	n/a
other_context	Context: other context	If the participant acquired English in a context that is not home, school, English-speaking country or informal	n/a	n/a
srep_s	Self-rated English proficiency: speaking	Self-rated English proficiency (scale 1 to 7) in speaking	n/a	1.3%
srep_r	Self-rated English proficiency: reading	Self-rated English proficiency (scale 1 to 7) in reading	n/a	2.7%
srep_w	Self-rated English proficiency: writing	Self-rated English proficiency (scale 1 to 7) in writing	n/a	1.4%
srep_l	Self-rated English proficiency: listening	Self-rated English proficiency (scale 1 to 7) in listening	n/a	1.3%
perc_eng	Use of English during the week	Percentage of time the participant used English over the course of a week	n/a	**4.4%**
Proficiency variable				
LexTALE_score	Objective English proficiency	Score obtained in the LexTALE test	n/a	**5.4%**

### Data cleaning

**Fig. 2:**
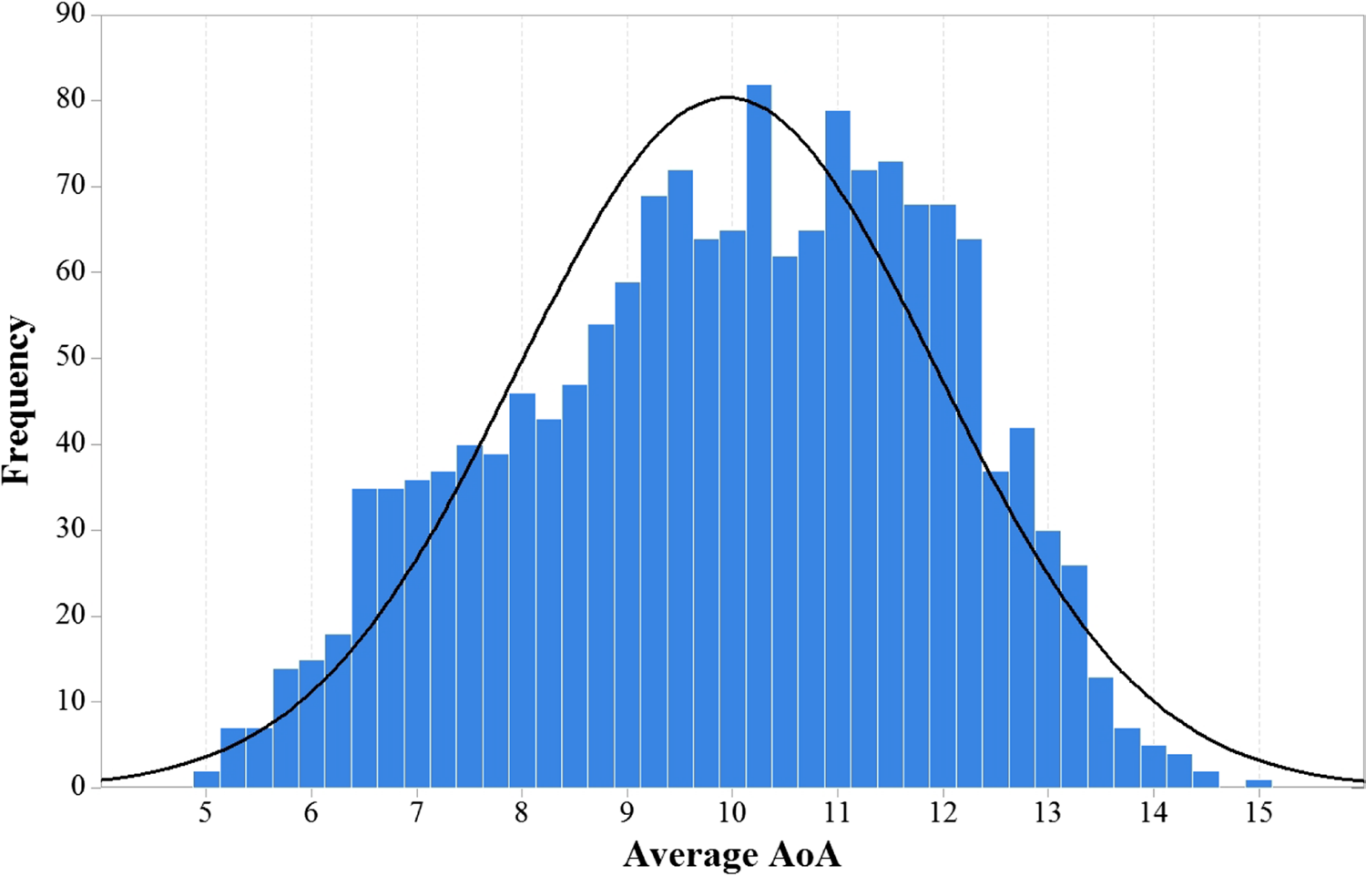
Frequency distribution of L2 AoA ratings

An initial sample of 309 participants took part in the study, but 292 remained after data trimming. Specifically, correlations between the responses of each participant and the mean responses of the other participants in the same questionnaire were computed to detect anomalous response patterns. Participants with a personal correlation with the mean of the group of less than 0.1 were discarded. This led to the exclusion of 17 participants (5.5% of the data). As observed in Fig. 2, the L2 AoA ratings data resemble those reported by Dirix and Duyck (2017) and Wang and Chen (2020).

### Reliability and validity

The inter-rater reliability was explored by calculating the intra-class correlation coefficient (ICC) for each AoA questionnaire with the *psych* package in R (Revelle, 2021), using the two-way random effects based on the absolute agreement of multiple raters (2, *k*). Data were strongly reliable (*M* =.93, *SD* = .017, range = .90–.96), even more so considering that L2 AoA data could bear more variability as L2 learning onset typically differs more between speakers than in L1 acquisition (see Dirix & Duyck, 2017).

**Fig. 3:**
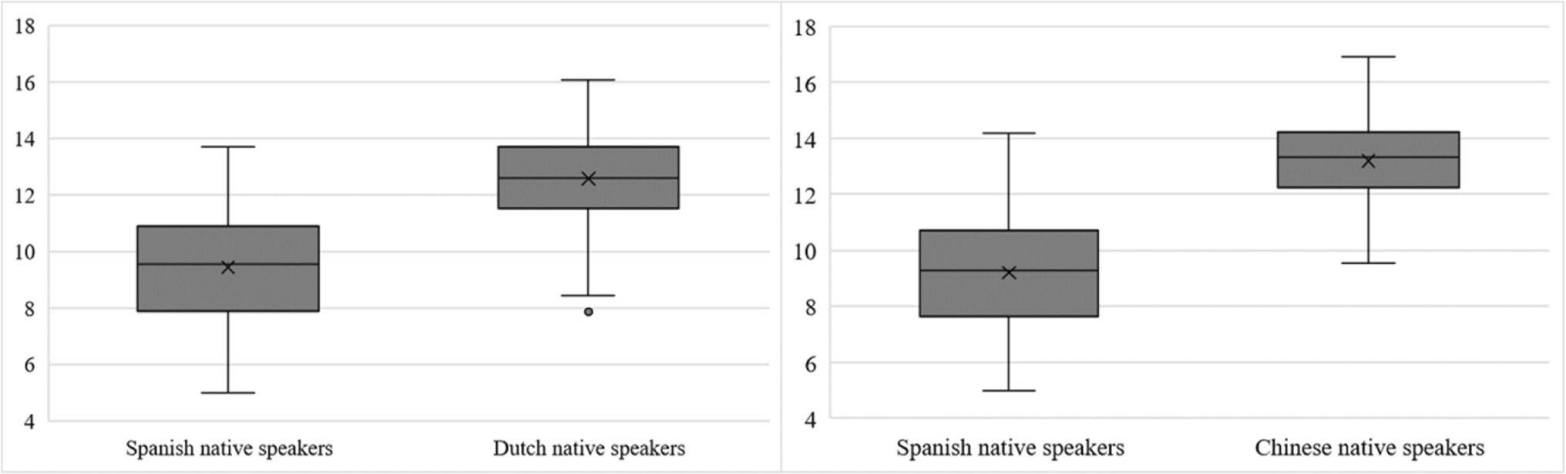
Left: boxplot displaying L2 AoA values in our database and Dirix and Duyck (2017), including the 549 words available in both databases. Right: boxplot displaying L2 AoA values in our database and Wang and Chen (2020), including the 731 words available in both databases

The validity of our ratings was assessed by performing Pearson’s correlations between our L2 AoA ratings and those provided by Chinese-English (Wang & Chen, 2020) and Dutch-English (Dirix & Duyck, 2017) speakers. Out of the 1835 words included in Wang and Chen (2020) and the 4900 words in Dirix and Duyck (2017), 731 and 549 were available in our database, respectively. The AoA values in our database showed a correlation of .78 (*p <* .001) with those from Wang and Chen (2020) and a correlation of .75 (*p <* .001) with the values of Dirix and Duyck (2017). Furthermore, we wanted to explore whether L2 AoA ratings followed a similar pattern in our database as in Dirix and Duyck (2017) and Wang and Chen (2020), by performing boxplots which indicate how the data are distributed. Data are shown in Fig. 3.

Our pattern of results differs from both Dirix and Duyck (2017) and Wang and Chen (2020). For the shared words in both databases, our average L2 AoA is lower (*M* = 9.43) and the dispersion in our sample is larger (*SD* = 1.96) than for the data obtained with native speakers of Dutch (*M* = 12.57; *SD* = 1.5). Something similar happens in comparison to the data of the native speakers of Chinese (*M* = 9.21, *SD* = 1.91 in our sample; *M* = 13.19, *SD* = 1.33 for the Chinese data). The results of two one-way analyses of variance (ANOVAs) (one comparing our data with those of Dirix & Duyck, 2017, and the other comparing our data with those of Wang & Chen, 2020) showed that those differences are significant. That is, the average L2 AoA estimate made by our participants (considering only the shared words between databases) is significantly lower than that estimated by native Dutch speakers, *F*(1, 1096) = 891.30, *MSE* = 2711.96, *p* < .001, *η*_p_^2^ = .45, and by native Chinese speakers, *F*(1, 1460) = 2130.02, *MSE* = 5786.04, *p* < .001, *η*_p_^2^ = .593.

We also correlated our L2 AoA ratings with L1 AoA ratings in English (Scott et al., 2019) and in Spanish (Alonso et al., 2015). All 1604 words were shared between our sample and Scott et al.’s (2019). In the case of Alonso et al. (2015), 1538 words (i.e., the Spanish translations of the English words) were shared between their database and ours. Our AoA values showed a correlation of .64 (*p <* .001) with the values from Scott et al. (2019), and a correlation of .58 (*p <* .001) with the values from Alonso et al. (2015) for Spanish-L1.

We also compared our L2 average AoA with the English L1 AoA values of Scott et al. (2019) and the Spanish L1 AoA values of Alonso et al. (2015, considering only the shared words between databases). The average AoA in our L2 sample is higher (*M =* 9.95) and more disperse (*SD* = 1.99) than the English L1 AoA data (*M* = 3.65; *SD* = 1.18). In comparison to the Spanish L1 AoA data, the average L2 AoA is higher (*M* = 9.91) but less disperse (*SD* = 1.99) than the Spanish L1 AoA data (*M* = 6.36; *SD* = 2.04). The average L2 AoA for English words was significantly higher in comparison to the English L1 data, *F*(1, 3206) = 11,887.82, *MSE* = 31,838.62, *p* < .001, *η*_p_^2^ = .788, and to the Spanish L1 data, *F*(1, 3074) = 2388.05, *MSE* = 9700.06, *p* < 001, *η*_p_^2^ = .437.

### Relationships between L2 AoA and lexico-semantic, affective, sociolinguistic and proficiency variables

**Fig. 4:**
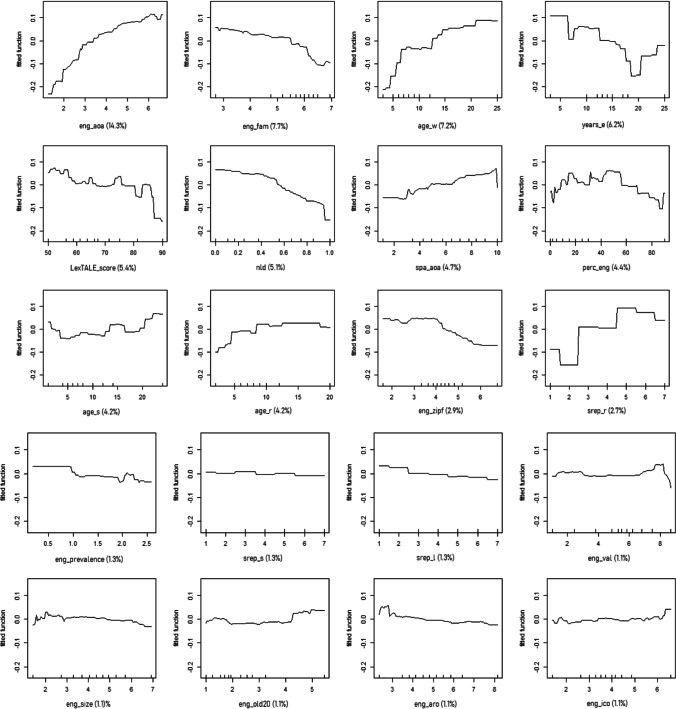

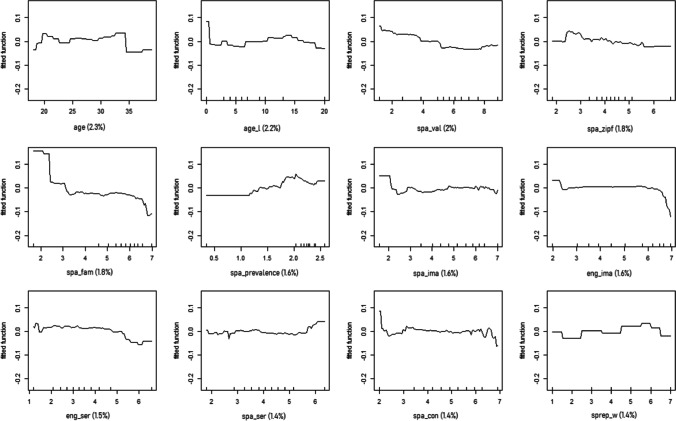
Partial dependence plots for the variables included in the simplified BRTs model predicting responses to the L2 AoA ratings, ordered by decreasing RC

Data were analysed using boosted regression trees (BRTs), which are an advanced form of regression analysis based on machine learning and boosting algorithms (Elith et al., 2008). It combines large numbers of simple tree models to optimise predictive performance by fitting many models and combining them for predicting the dependent variable. Also, and unlike other regression methods, BRTs use an algorithm to learn the relationship between predictors and responses, instead of setting up a model first and then estimating parameters for the model from the data. This implies that BRTs considers that the relationships between predictors and responses are unknown and tries to learn about this relationship by processing inputs and responses and finding dominant patterns. BRTs also identify linear and non-linear relationships, which can be observed on the generated graphs (see Fig. 4). Raw data are available at https://osf.io/gr8xd/?view_only=73b01dccbedb4d7897c8d104d3d68c46

Following the guidelines established by Elith et al. (2008) and Elith and Leathwick (n.d.), we first fitted a model including all predictors, using a tree complexity of 5, a learning rate of 0.01 and a bag fraction of 0.5. This model accounted for a relevant proportion of deviance (58.75%). Then, we simplified the model by dropping predictors until the average change in predictive deviance exceeded their original standard error. The simplified model eliminated 14 out of the 46 predictors, given their low predictive value: English_country, eng_length, difficulty, school, spa_length, home, sex, education, informal, spa_old20, eng_con, other_context, spa_aro and spa_ico. Also, the simplified model accounted for a relevant proportion of deviance, virtually identical to that of the complete model (58.56%, see Fig. 4). The predictors included in the simplified model and their relative contributions (RCs) can be found in Table 2. Below, data will be presented in three subsections: first, variables related to the words (lexico-semantic and affective), then, variables related to the speakers (sociolinguistic and proficiency), and finally, L2 AoA values and their relationship with L2 reaction times.

### L2 AoA and lexico-semantic and affective variables

All the RCs of the predictors can be found in Table 2. The predictor with the highest RC was eng_aoa (14.3%), showing that the earlier the English L1 AoA, the earlier the L2 AoA. Then, other predictors showed a moderate RC: eng_fam (7.7%, the higher the familiarity, the earlier the L2 AoA), nld (5.1%; the smaller the Levenshtein distance between English and Spanish words, the earlier the L2 AoA) and spa_aoa (4.7%; the earlier the Spanish AoA, the earlier the L2 AoA). The other predictors had a smaller RC (<3%, see Fig. 4 for more information).

### L2 AoA and sociolinguistic and proficiency variables

All the RCs of the predictors can be found in Table 2. The predictor with the highest RC was age_w (7.2%), showing that the earlier the age of writing in English, the earlier the L2 AoA. Other predictors showed a moderate RC: years_e (6.2%; non-linear relationship, although, in general, the more time participants spent learning English, the later their L2 AoA), LexTALE_score (5.4%; the higher the proficiency, the earlier the L2 AoA, becoming more acute at high proficiency levels), perc_eng (4.4%; non-linear relationship), age_s (4.2%; non-linear relationship) and age_r (4.2%; the earlier the participants started reading in English, the earlier the L2 AoA, although this is more pronounced for early ages). The other predictors had a smaller RC (<3%, see Fig. 4 for more information).

### L2 AoA and L2 reaction times

The relationship between L2 AoA values and L2 RTs in native Spanish L2 speakers of English was explored by retrieving 1089 words from Berger et al. (2019). We correlated our L2 AoA data with the L2 RT data from Berger et al. (2019). Results were *r* = .207, *p* < .001, which supports a positive influence of L2 AoA on L2 RTs (i.e., the earlier an L2 word is learned, the faster speakers identify it, and vice versa). In addition, we correlated L2 AoA values and L2 RTs using partial correlations that controlled for the potential influence of AoA in Spanish (values were extracted from Alonso et al., 2015 and were available for 1049 words) and showed that L2 AoA continued to be a reliable predictor, *r* = .189, *p* < .001. We also correlated the Spanish AoA data with the L2 RT data for Spanish-English speakers, controlling for L2 AoA, finding *r* = −.027, *p* = .391, showing that L1 AoA did not exert any influence on L2 RTs.

## Discussion

In the current article, we present a database of subjective L2 AoA ratings for 1604 English words provided by Spanish L2 speakers of English. It provides more ecological data for psycholinguistic studies with Spanish L2 speakers of English. In addition, we wanted to examine whether, and how, several variables related, on the one hand, to the words (both lexico-semantic and affective variables), and, on the other hand, to the participants (sociolinguistic and proficiency variables), modulated the AoA ratings. For the database, we gathered the ratings from 292 participants, and we make it available to the research community. Our L2 AoA ratings follow the pattern of previous studies, such as those of Dirix and Duyck (2017) or Wang and Chen (2020). Our results were highly correlated with these other databases, which supports the validity of our data. Specifically, high correlations were found between our ratings and those reported in other studies looking at subjective L2 AoA (Dirix & Duyck, 2017; Wang & Chen, 2020). Our ratings were also correlated, to a slightly lesser extent, with L1 AoA ratings in English (Scott et al., 2019) and Spanish (Alonso et al., 2015). Therefore, we are confident we are offering a solid resource for L2 psycholinguistic research.

Regarding the exploration of how word- and participant-related factors modulated AoA ratings, 46 predictors were included in the BRTs analyses. Our results showed that AoA in English was the factor that contributed the most to the simplified model; in other words, the age at which native English speakers acquire certain words could predict the age at which non-natives acquire those words as well. Below, we discuss some further results that may be of interest.

Following previous literature, we stated a few initial hypotheses. First, we expected to find a positive relationship between L1 (Spanish) AoA and L2 AoA scores. This was motivated by previous literature gathering L2 AoA ratings, where both Dirix and Duyck (2017) and Wang and Chen (2020) found a moderate positive correlation between the AoA of L1 and L2 words in their studies. We also found a moderate correlation with the L1 AoA data gathered by Alonso et al. (2015), and additionally found L1 AoA to be a relevant predictor in the BRTs simplified model. Therefore, it seems that the role of L1 AoA is undeniable for L2 AoA (at least, when English is the L2), regardless of the L1 of the speakers. Also, it is remarkable that our L2 AoA data correlated more highly with other L2 AoA data (i.e., the Dutch-English and Chinese-English data by Dirix & Duyck, 2017, and Wang & Chen, 2020, respectively) than with L1 AoA data in Spanish or English. Although this would require further examination, it seems that, regarding AoA, the acquisition of words in an L2 follows a similar pattern for L2 speakers from different backgrounds, with more similarity between L2 AoAs in different languages than between the AoA in L2 and the AoA of the two languages of an L2 speaker, which in our case is Spanish and English. However, differences in the pattern of L2 AoA are also noticeable between our data and the previous work by Dirix and Duyck (2017) and Wang and Chen (2020). The data shown in the boxplots (Fig. 3) and the results from the ANOVAs illustrate two main findings: (1) Spanish natives acquire L2 words earlier, and (2) AoA scores in L2 from Spanish natives show more variability than those from Dutch and Chinese natives. To account for these differences, we would need to consider educational and social factors. In Spain, a foreign language is learned at school from the first year of compulsory education, that is, from 6 years of age (LOMLOE, 2020). English formal education for the Dutch sample is reported to start at age 13 in the case of Dirix and Duyck (2017), and Wang and Chen (2020) declared that most of their participants started learning English at 7 to 9 years of age. Therefore, at least in terms of formal education, participants have different starting points.^3^

Secondly, a negative relationship between word familiarity in English and L2 AoA was expected, meaning that more familiar words would be acquired earlier, in line with Wang and Chen (2020). Our data showed that familiarity of the English words was the second most relevant predictor in the simplified model, only after the AoA of the English word. Also, as can be observed in Fig. 4, this relationship is negative: the higher the familiarity, the earlier the word was acquired in the L2. Therefore, our results are in line with those from Wang and Chen (2020), even though they collected familiarity scores provided by their Chinese L2 English speakers, while we collected familiarity ratings in English provided by native speakers from the Glasgow norms (Scott et al., 2019), and not from our participants.

Our third objective was to explore whether, and how, some variables linked to the speaker (i.e., sociolinguistic variables and proficiency) could affect L2 AoA ratings. In a study where valence and arousal ratings for L2 words were collected, Imbault et al. (2021) found that participants with certain characteristics provided more native-like ratings. These features included (a) learning English at a young age, (b) for a long number of years, and (c) in an immersive context; (d) having high self-reported proficiency and (e) using English frequently in their day-to-day lives (Imbault et al., 2021). These five variables were included in our model. “Learned English at a young age” was assessed through four variables, namely age at which English was acquired for speaking, reading, writing and listening. We found that all these variables contributed to the model, with the age at which English was acquired for writing having the largest RC, followed by speaking and reading, and finally listening. “Learning English for a long number of years” would be equivalent to “number of years that they have been studying English”, which was the fourth most relevant variable in the model. It is important to note that in this case, our data show a non-linear relationship where learning for more years does not always (i.e., around 6 and 18–19 years; see Fig. 4) involve earlier AoA ratings. “Learning English in an immersive context” does not have an equivalent in our study, but it could be inferred from variables such as “learning English at home” or at an “English-speaking country”; however, these two variables were not selected for the simplified model given their low RC. “High self-reported proficiency” was assessed in this case with self-rated proficiency for speaking, reading, writing and listening, which entered the model, with self-reported proficiency for reading having the largest RC, followed by writing, and speaking and listening (although the RC for all these variables was rather small). Finally, the percentage of use of English also contributed to the model, and, in addition, it correlated positively with the speakers’ proficiency. In sum, our results support the notion that sociolinguistic variables influence L2 ratings, extending to L2 AoA what Imbault et al. (2021) found for L2 valence and arousal.

Our study also identified other variables that contribute to AoA L2 ratings. Considering that 32 predictors entered the simplified model, we would like to comment on those that had larger RCs, presenting them separately in variables related to the words (i.e., lexico-semantic and affective) and to the participants (i.e., sociolinguistic and proficiency). Regarding those linked to the words, after AoA in English and English familiarity, the variables that had a larger RC were cognate status and frequency in English (please note that a negative relationship between AoA and frequency—that is, the higher the frequency, the lower the AoA—seems to arise mostly for medium- to high-frequency scores). Our results follow the same trends as those of studies such as Tonzar et al. (2009) and Comesaña et al. (2012), who showed that cognates facilitate L2 acquisition, and of others like Citron et al. (2014), who reported that high-frequency words are also acquired earlier (frequency in Spanish also contributed to the model but to a lesser extent than frequency in English). With a smaller contribution, we found other results agreeing with previous literature (although these data should be taken with caution, as the RCs for these predictors were rather small). For instance, the contribution of valence in Spanish and valence in English (with a smaller RC value) agrees with Citron et al. (2014), Hinojosa et al. (2016a) and Warriner et al. (2013), and indicates that at young ages, people tend to prefer positive stimuli or overestimate the positive valence of stimuli, as observed by previous studies with children (see, for instance, Ponari et al., 2018; Sabater et al., 2020; Sylvester et al., 2016). We have also found an influence on our L2 AoA ratings of prevalence of Spanish words, and to a lesser extent, of English words (supporting Brysbaert et al.’s findings, 2009, 2016), imageability in Spanish and English (in line with Citron et al., 2014) and of SERs both in English and Spanish (agreeing with Hinojosa et al., 2016b). However, it cannot be said that our results are conclusive, because the relationships that we have found for these variables are not linear. Also, results are inconclusive for the rest of the lexico-semantic and affective variables entering the simplified model, either because they depict a non-linear relationship or because a particular variable enters the model for one language but not for the other (i.e., concreteness in Spanish, iconicity in English, arousal in English).

As for those factors related to the participants, the most relevant variable seems to be proficiency, as more proficient speakers assigned lower AoA scores (where this is accentuated for very proficient speakers).

Finally, we aimed at exploring the relationship between L2 AoA and L2 processing by retrieving L2 RT data from a previous independent study (i.e., Berger et al., 2019). This was motivated by Izura and Ellis’s (2002) work. The authors looked at the relationship between the AoA of the speaker’s first and second language, and the RTs in language processing tasks, adding to the theoretical discussion regarding the locus of the AoA effect. They found that L2 AoA does not correspond with the order in which the equivalent word meanings were acquired in the L1. Following Izura and Ellis (2002), if L2 AoA depended on L1 AoA (i.e., in the process of acquiring a word in the L2, this new word form would be associated with an old semantic representation), the source of the AoA effects would lie in the semantic system, so the age at which the concept was firstly acquired (that is, in the L1) would determine RTs in the L2 too. In practical terms, the AoA of the speakers’ L1 (in this case, Spanish) should positively correlate with L2 RTs. However, if the locus is not in the semantic system and were to lie in the mapping between lexical and semantic representations (as predicted by the *mapping hypothesis*), L2 AoA, but not L1 AoA, should positively correlate with L2 RTs. The latter was found by Izura and Ellis (2002), concluding that the L2 AoA effect mirrors the order in which the words were acquired in the L2, rather than the order in which the translation equivalents were acquired in the native language. We have been able to replicate Izura and Ellis’ (2002) findings with a larger dataset, adding to the theoretical discussion of the locus of the AoA effect by advocating in favour of the *mapping hypothesis* and against a semantic locus for this effect.

At this point, some remarks are needed. First of all, this is the first time, to our knowledge, in which BRTs are used to explore word-related and participant-related data on AoA. As pointed out above, BRTs enable one to explore the relationship between variables without prior assumptions, allowing researchers to establish patterns in their data that might not have been explored if following other types of analysis. Also, previous research tends to consider only a few of these variables as predictors, but we included 32 predictors in our simplified model (considering data in English and Spanish). On top of that, what we know about the influence of these lexico-semantic and affective variables for AoA is limited to AoA ratings provided by native speakers, so the hypotheses that can be derived using these norms are limited as well. Lastly, it must be kept in mind that the lexico-semantic and affective values that we have employed in the analyses carried out here have been normed by L1 speakers. Therefore, in order to clearly elucidate which lexico-semantic and affective variables influence L2 AoA, it would be beneficial to use ratings normed by L2 speakers, who ideally share a native language.

Finally, we would like to highlight the relevance of including variables related to the words (lexico-semantic and affective) and to the speakers (sociolinguistic and proficiency) in this sort of research. The results of our model show that a combination of these factors is key to explaining L2 AoA, where if we were to overlook, for instance, the role of the variables related to the speakers, we would have lost valuable information. Therefore, in our quest to make L2 research more ecologically valid, we need to include not only ratings performed by the type of speaker that we are going to investigate (that is, ratings by and for L2 speakers), but also information about sociolinguistic factors that indeed shape L2 acquisition.

## Conclusions

In the current article, we provide the research community with an L2 AoA database by and for Spanish-English speakers. We also carefully examined the relationship between lexico-semantic, affective, sociolinguistic and proficiency variables and L2 AoA, finding that a combination of these variables is needed to obtain comprehensive models of L2 AoA. Our results also support the *mapping hypothesis* regarding the locus of the AoA effects. We hope that our contributions prove useful for researchers involved in psycholinguistic research with Spanish L2 speakers of English and can ultimately be informative for second language teaching or bilingual education programmes.

## Footnotes

1 We performed a correlational analysis on the mean AoA and familiarity ratings provided in the supplementary materials by Hinojosa et al. (2016b) showing that words acquired earlier were also more familiar (*r* = −.665, *p* < .001).

2 We performed a correlational analysis on the mean AoA and SERs provided in the supplementary materials by Hinojosa et al. (2016b) showing that words acquired earlier have higher SERs (*r* = −.255, *p* < .001).

3 As one reviewer suggested, we explored whether our lower AoA average is due to population differences, as we propose, or whether it could respond to the fact that we provided a top threshold (i.e., +15) in our study, whereas Dirix and Duyck (2017), and Wang and Chen (2020) did not. To examine this issue, we selected those words in our database for which participants never chose the 15+ option (*n* = 412) and compared them with the corresponding words available in the Dutch database (*n* = 188) and the Chinese database (*n* = 263). The AoA values showed a correlation of .657 (*p* < .001) with those from Wang and Chen (2020) and a correlation of .663 (*p* < .001) with the values of Dirix and Duyck (2017). For shared words in both databases, our average L2 AoA is lower (*M* = 7.52) and the dispersion in our sample is smaller (*SD* = 1.14; contrary to when using the full dataset) than for the data obtained with native speakers in Dutch (*M* = 11.45, *SD* = 1.17). Something similar happens in comparison to the data of native speakers of Chinese (*M* = 7.52, *SD* = 1.15 in our sample; *M* = 12.29, *SD* = 1.18 for the Chinese sample). The results of two one-way ANOVAs (one comparing our data with those of Dirix & Duyck, 2017; and other comparing our data with those of Wang & Chen, 2020) showed that those differences are significant. That is, the average L2 AoA estimate made by our participants (considering only the shared words between databases) is significantly lower than that estimated by Dutch native speakers, *F*(1, 374) = 1087.68, *MSE* = 1.334, *p* < .001, *η*_p_^2^ = .74, and by Chinese native speakers, *F*(1, 524) = 2193.32, *MSE* = 1.362, *p* < .001, *η*_p_^2^ = .81. In sum, the pattern of results does not change substantially when eliminating the +15 words, which speaks in favour of the population differences.
